# PGC-1α controls mitochondrial biogenesis and dynamics in lead-induced neurotoxicity

**DOI:** 10.18632/aging.100790

**Published:** 2015-09-04

**Authors:** Aleksandra Dabrowska, Jose Luis Venero, Ryota Iwasawa, Mohammed-khair Hankir, Sunniyat Rahman, Alan Boobis, Nabil Hajji

**Affiliations:** ^1^ Imperial College London, Centre for Pharmacology and Therapeutics, Department of Medicine, London, United Kingdom; ^2^ Departamento de Bioquímica y Biología Molecular. Facultad de Farmacia, Universidad de Sevilla, C/Prof. García González, Sevilla, Spain; ^3^ Integrated Research and Treatment Centre for Adiposity Diseases, Department of Medicine, University of Leipzig, Leipzig, Germany

**Keywords:** neurotoxicity, Lead, mitochondrial biogenesis and dynamics, PGC-1α, Drp1, BAP3, calcium

## Abstract

Due to its role in regulation of mitochondrial function, PGC1α is emerging as an important player in ageing and neurodegenerative disorders. PGC1α exerts its neuroprotective effects by promoting mitochondrial biogenesis (MB) and functioning. However, the precise regulatory role of PGC1α in the control of mitochondrial dynamics (MD) and neurotoxicity is still unknown. Here we elucidate the role of PGC1α *in vitro* and *in vivo* in the regulatory context of MB and MD in response to lead (II) acetate as a relevant model of neurotoxicity. We show that there is an adaptive response (AR) to lead, orchestrated by the BAP31-calcium signalling system operating between the ER and mitochondria. We find that this hormetic response is controlled by a cell-tolerated increase of PGC1α expression, which in turn induces a balanced expression of fusion/fission genes by binding to their promoters and implying its direct role in regulation of MD. However, dysregulation of PGC1α expression through either stable downregulation or overexpression, renders cells more susceptible to lead insult leading to mitochondrial fragmentation and cell death. Our data provide novel evidence that PGC1α expression is a key regulator of MD and the maintenance of tolerated PGC1α expression may offer a promising strategy for neuroprotective therapies.

## INTRODUCTION

Peroxisome proliferator-activated receptor-γ coactivator-1α (PGC-1α) is a transcription factor controlling many aspects of oxidative metabolism, including mito-chondrial biogenesis, adaptation, respiration, adaptive thermogenesis, gluconeogenesis and oxidative phosphorylation [1, 2]. Signalling for mitochondrial biogenesis (MB) is activated by PGC-1α and involves the expressionof several transcription factors, resulting in the upregulation of proteins encoded by both nuclear and mitochondrial genomes. Oxidative stress from the generation of reactive oxygen species (ROS) has often been cited as toxicants, and is believed to originate from mitochondria. Many neurodegenerative conditions have been shown to originate from compromised number, morphology and function of mitochondria. PGC1α has previously been reported to play a protective role against neurodegenerative conditions such as Alzheimer's disease and is known to respond to ROS by the induction of many ROS-detoxifying enzymes, including superoxide dismutase, Gpx1and γ-glutamylcysteine, regulating the biosynthesis of glutathione. Moreover, Ca^2+^ and ROS have been shown to regulate mitochondrial biogenesis by activating PGC1α, leading to an increase in mitochondrial mass [3, 4].

Growing evidence suggests that the delicate equilibrium between mitochondrial fission and fusion is vital for many mitochondrial functions including metabolism, energy production, Ca^2+^ signalling, ROS production and apoptosis. In many types of neurodegenerative diseases, a significant reduction in the levels of fusion proteins optic atrophy-1 (Opa-1), mitofusin-1 (Mfn-1) and mitofusin-2 (Mfn-2) as well as the fission protein dynamin-related protein-1 (Drp-1) is reported, as well as an increase in the level of the fission related protein-1 (Fis-1) [5].

A number of signalling processes relating to mitochondrial, nuclear and inter-organelle communica-tion, have been shown to be sensitive to ROS. Upon oxidative stress, the mitochondria and the endoplasmic reticulum critically contribute to apoptosis induction via a signalling pathway that establishes a feedback loop by releasing Ca^2+^ from the ER which in turn activates the mitochondria for apoptosis [6].

The majority of mechanisms proposed for neurotoxicant-induced cellular damage converge on mitochondrial dysfunction and energy deprivation [7]. The integrity of this organelle is vital in the context of neurodegeneration due to the high-energy demands of dopaminergic neurons as a result of their unusually long axons [8]. This generates a need for the stringent regulation of mitochondrial biogenesis, function and dynamics in such cells. Disruption of any of these processes can contribute to neurodegenerative disorders [9] such as Parkinson's [10], Alzheimer's [11] and Huntington's disease [12]. However, no study has fully elucidated a link between mitochondrial biogenesis and dynamics in neurotoxicity, highlighting the need for a thorough investigation into PGC1α's role in those processes in the context of neurological disorders.

Chronic exposure to the environmental neurotoxicant lead is believed to be associated with neurodegeneration [13]. While it has been proposed that there is an association between chronic lead exposure and risk of Parkinson's disease (PD) [14, 15], the exact mechanism is not fully understood. Exposure to lead induces oxidative stress in the form of ROS in a variety of different tissues [16, 17] and also induces Ca^2+^ release from the ER in hippocampal neurons [18]. However, evidence is lacking with regards to the mechanism of neuronal damage and whether its induction in chronically exposed individuals is due to an increased production of ROS or disruption of calcium homeostasis or both.

In this study we investigated the role of PGC1α as a regulator of both mitochondrial biogenesis and dynamics *in vitro* and *in vivo* in the context of lead-induced neurotoxicity. Strikingly, the role of PGC1α was found to be equivocal. While a modest increase of PGC1α expression provides a neuroprotective role, dysregulation of expression either by knockdown or overproduction is non-tolerated, leading to neurotoxicity. Our data showed a significant increase of expression of a mitochondrial fission protein, DRP1, in both,neuronsin the rat substantia nigra (SN) and adopaminergic (DA) neuronal N27 cell line. SN cells showed an adaptive response to lead exposure by increasing the transcript levels of PGC1α, its target genes, regulating mitochondrial biogenesis, and mitochondrial fusion- and fission-related genes. ChIP and QPCR analysis revealed that PGC1α directly regulates DRP1expression by binding to its promoter. Our data provides strong evidence that PGC1α is directly involved in the cellular response to lead by regulating mitochondrial dynamics.

## RESULTS

### Exposure to lead induces mitochondrial dysfunction, energy depletion and subsequent apoptosis of dopaminergic neurons

**Figure 1:**
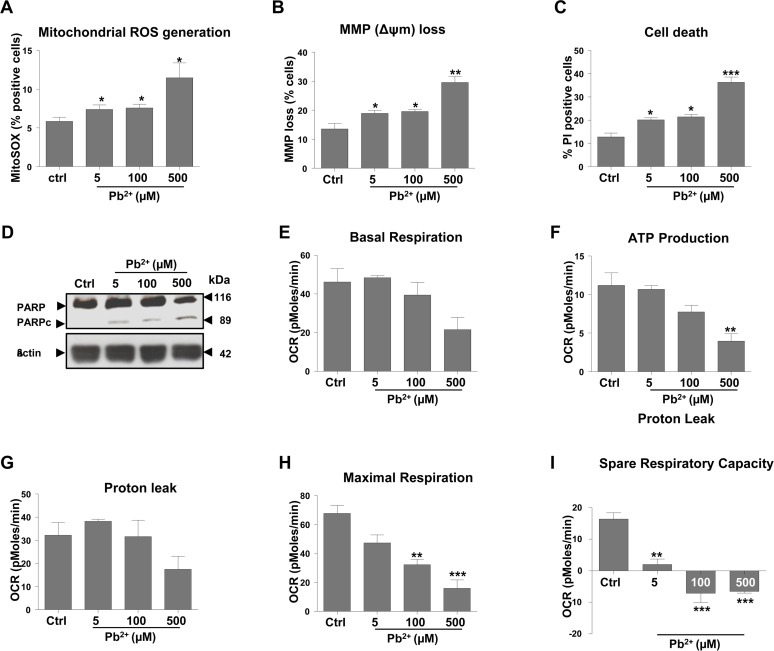
Exposure to lead induces mitochondrial dysfunction and subsequent apoptosis of N27 dopaminergic neurons Cells were incubated for 48-hours with lead acetate, at the concentrations shown. (**A**) Mitochondrial superoxide levels were measured using MitoSOX staining followed by FACS analysis. (**B**) The effect of lead treatment on mitochondrial membrane potential (MMP) loss was detected by DiOC_6_(3). (**C**) Analysis of cell death measured by propidium iodide (PI). (**D**) Western blot analysis of PARP cleavage (PARP to PARPc) following lead exposure. Profiles of different parameters of mitochondrial function (**E-I**) were determined using Seahorse XF24 Analyser. *P<0.05, **P<0.01, ***P<0.001, n=3; mean ± SE.

N27 cells, derived from rat mesencephalon, were used to evaluate the effect of lead on mitochondrial function. Cells were treated with a range of lead (II) acetate concentrations over a period of 48 hours. Cells stained with MitoSOX and analyzed by flow cytometry revealed a 25% increase in mitochondrial superoxide at 5 μM and 100 μM concentrations and almost a 100% increase at 500 μM (Fig. 1A). DiOC_6_(3), a selective mitochondrial dye, and propidium iodide (PI) staining revealed that lead causes mitochondrial membrane depolarization and neuronal cell death, which was much more marked at 500 μM than at 5 or 100 μM (Fig. 1B, C). PARP cleavage analysis (Fig. 1D) confirmed this, with the effect being far more pronounced at the highest lead concentration. At 5μM and 100μM, effects on mitochondrial dysfunction and cell death were similar (Fig. 1A-D). Apoptotic cell death was confirmed by an increased activation of caspases3/7 and caspase 8 in N27 cells exposed to 100μM and 500μM lead (Supplementary Figure1-S-1, A-a and b). Morphological characteristics of apoptotic cells, including condensed nuclei, were also evident in cells treated with 500μM lead (Supplementary Figure1-S-1, B and C). Morphological changes in the cells were apparent only at higher concentrations than changes in molecular markers of apoptosis, most likely reflecting the temporal engagement of the respective hallmarks of apoptosis.

Mitochondrial function was assessed by *Seahorse XF24* analysis allowing for the measurement of a number of parameters including mitochondrial basal respiration, ATP production, oxygen consumption rate (OCR), maximum respiratory capacity and mitochondrial spare respiratory capacity. While a reduction in basal respiration was observed in cells treated with 100 and 500 μM lead (Fig. 1E), this was not statistically significant. There was a concentration-dependent decrease in ATP production (Fig. 1F), with the highest lead concentration causing a statistically significant (P<0.01) decrease of more than 50%. Proton leak was not significantly affected by lead treatment (Fig. 1G). Maximal respiration decreased in a concentration-dependent manner (Fig. 1H). Interestingly, mitochondrial reserve capacity levels were very low across all of the lead-treated cells (P<0.01) including 5 μM, a concentration which did not have a significant impact on other aspects of mitochondrial function investigated with the Seahorse XF24 compared with control (Fig. 1I).

### PGC1α protects DA neurons from neurotoxic insults from lead

**Figure 2:**
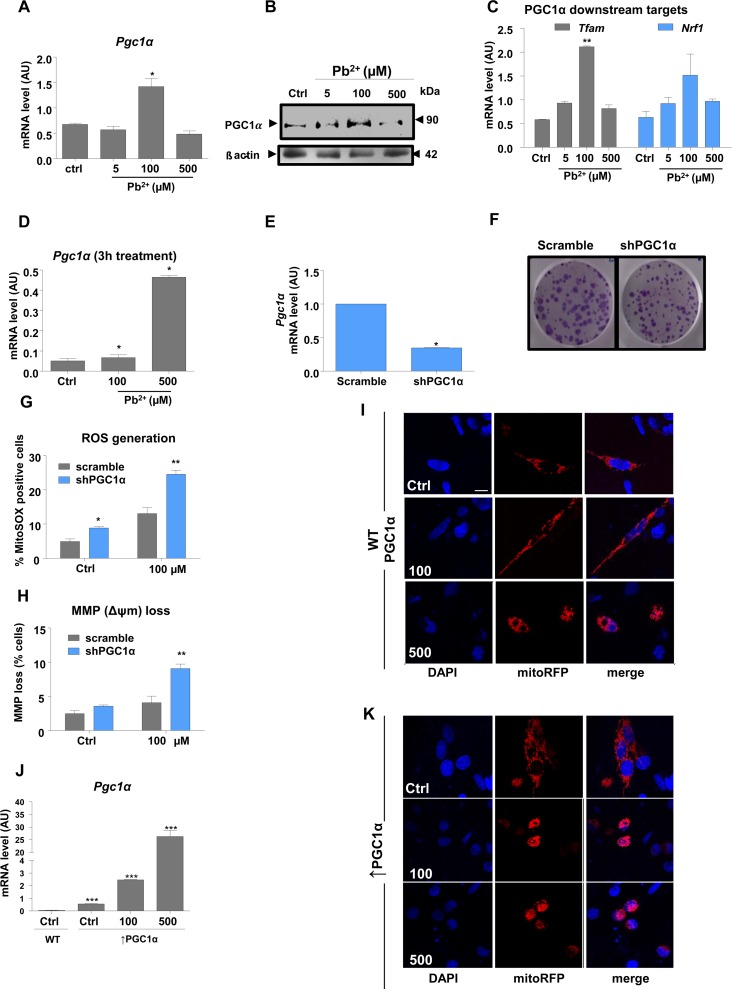
PGC1α protects N27 dopaminergic neuronal cells from Pb^2+^-induced neurotoxicity Cells were incubated with lead acetate at the concentrations shown. (**A**,**B**) qRT-PCR and western blotting measurement of mRNA and protein expression levels, respectively of *Pgc1α* in N27 cells treated with lead for 48-hours. (**C**) mRNA levels of *Pgc1α* target genes *(Tfam, Nrf1)*inN27 cells treated with lead for 48-hours.(**D**)N27 *Pgc1α*mRNA levels after 3-hours lead treatment.(**E**) *Pgc1α* mRNA levels and (**F**) colony formation in N27 cells with stable downregulation of PGC1α. (**G**) Mitochondrial superoxide levels were measured in shPGC1α cells using MitoSOX staining and (**H**) the effect of lead treatment on mitochondrial membrane potential loss in shPGC1α was detected by DiOC_6_(3) in N27 cells treated with lead for 48-hours. (**I**,**K**) Confocal imaging analysis of mitochondrial morphology in wild type PGC1α and in overexpressed PGC1α N27 cells ((*Pgc1α*) was investigated using Mito Tracker and DAPI staining recorded by fluorescence microscopy. (**J**) qRT-PCR analysis of *Pgc1α* in N27 cells expressing exogenous *Pgc1α* versus(*Pgc1α* cells, treated with lead for 48-hours.*P<0.05, **P<0.01, n=3; mean ± SE.

Real-time QPCR and protein expression analyses were performed to investigate the expression of *Pgc1α* and its downstream targets, which regulate mitochondrial biogenesis, in lead-treated N27 cells. Interestingly, only the 100 μM treatment with lead for 48 h caused a significant increase of *Pgc1α* expression, at both the RNA and protein levels (Fig. 2A, 2B). Downstream targets of PGC1α, *Tfam* and *Nrf1* showed a similar pattern of expression, with changes apparent only at 100 μM lead (Fig. 2C). In order to determine whether the highest lead concentration affects *Pgc1α* expression at an early exposure time point, *Pgc1α* mRNA levels were investigated after a 3-hour lead exposure. Surprisingly, the expression of *Pgc1α* mRNA levels was markedly upregulated 3-hour post-treatment, with a ten-fold increase observed at 500 μM (Fig. 2D).

Since no significant difference was observed between cells treated with 5 μM and 100 μM in terms of disruption of mitochondrial function, ROS generation or cell death(Fig. 1A-D), but there was a significant difference in *Pgc1α* mRNA levels between the two groups, we sought to investigate whether *Pgc1α* plays a protective role against lead insult at 100 μM. To interrogate this hypothesis, we produced a stable knock-down of *Pgc1α* using a lentiviral shRNA vector. Successful knock-down was confirmed by *Pgc1α* mRNA levels(Fig. 2E) and clear downregulation of PGC-1α protein levels, as shown by immunocyto-chemistry (Supplementary Figure3-S3, A). sh-PGC1α transduction did not affect colony formation (Fig. 2E and F). FACS analysis of cells stained independently with MitoSOX and DiOC_6_(3) showed that reduction of *Pgc1α* expression renders N27 cells more susceptible to lead-induced mitochondrial stress (Fig. 2G, H), with morphological characteristics of apoptosis apparent in cells treated with 100μM or 500μM lead (Supplementary Figure1-S1, B).

The influence of lead treatment on mitochondrial organisation was investigated by immunofluorescence (Fig. 2I). Exposure to 100 μM lead caused mitochondrial filament elongation, while mitochondria in cells exposed to 500 μM Pb^2+^ were very fragmented and localized to the nuclear perimeter (Fig. 2I).

### An equivocal role of PGC1α in neuroprotection and toxicity

**Figure 3:**
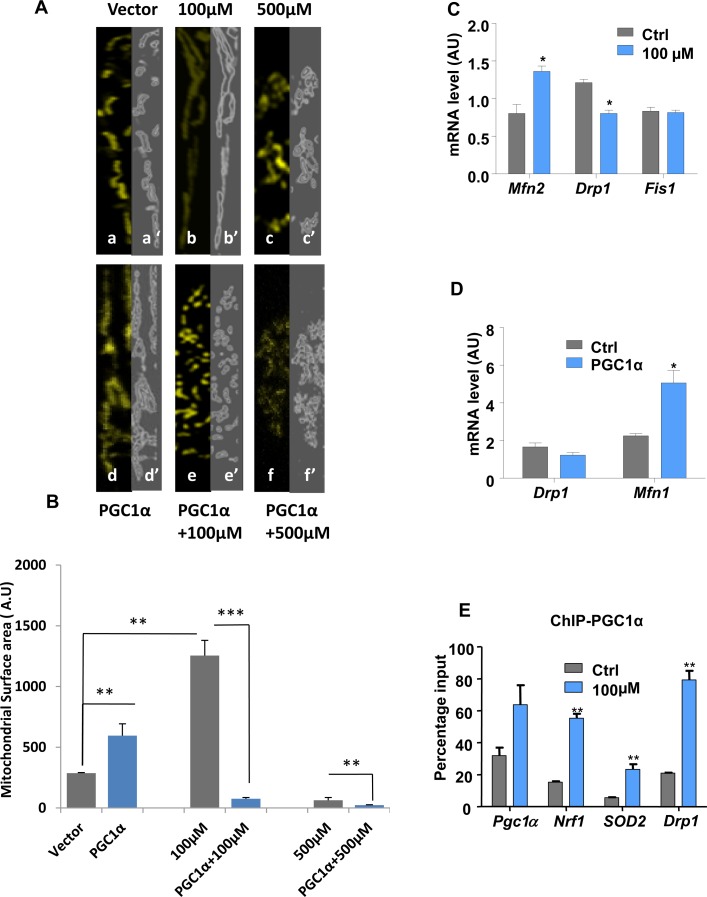
Lead-induced changes in mitochondrial morphology and dynamics Immunocytochemistry and confocal imaging analysis of N27 cells co-transfected with an empty vector and mitoYFP (**A**, a-c) or PGC1α-encoding vector and mitoYFP (**A**, d-f). The extent of the mitochondrial network both under basal conditions (a), Pb^2+^ 100μM (b), Pb^2+^ 500μM (c) and PGC1α overexpression alone (d) or combined with Pb^2+^ 100μM (e) or 500 μM (f) was measured by Spectrum. (**A**, a’-f’) mitochondrial area surface determined using ImageJ->threshold->Intermodes,(B,W). (**B**) Mitochondrial surface area in cells was determined using ImageJ software.(**C**) N27 cells treated with 100μM lead for 48-hours were analysed for the expression of mitochondrial fusion (*Mfn2*) and fission (*Drp1, Fis1*) genes by qRT-PCR. (**D**) qRT-PCR analysis of the expression of *Drp1* and *Mfn1* in PGC1α overexpressing cells.(**E**) Chromatin immunoprecipitation (ChIP) with a PGC1α antibody was performed to investigate PGC1α protein binding to its promoter, Nrf1, Drp1 and SOD2 gene promoters,*P<0.05, **P<0.01, ***P<0.001, n=3; mean ± SE.

To determine whether up-regulation of PGC1α was responsible for the changes in mitochondrial morphology and organization and subsequent protection of DA neurons from lead insult, we examined the effects of PGC1α overexpression on the structure of mitochondria (Fig. 2J and K) and (Fig. 3A and B). Intriguingly, overexpression of PGC1α actually increased susceptibility of N27 cells to lead toxicity at both 100 and 500 μM, as manifested by increased fragmentation of mitochondria, measured as a decrease in mitochondrial surface area (Fig. 3A and B). Whereas a two-fold increase in PGC1α expression protected cells against lead-induced neurotoxicity (endogenous levels of the protein doubled in response to 100 μM lead), higher expression levels (>50 fold) of the protein had the opposite effect and exacerbated the toxicity of lead (Fig. 2J and K). Furthermore, cells overexpressing PGC1α treated with 100μM and 500μM leaded showed condensed nuclei, an apoptotic hallmark (Supplementary Figure1-S1, C).

Since exposure to lead induced changes in mitochondrial organization, the expression profiles of genes encoding proteins regulating mitochondrial dynamics were investigated. RT-QPCR analysis revealed that 100 μM lead shifts the mitochondrial dynamic profile towards fusion (Fig. 3C). While *Mfn2* mRNA levels were increased, the transcript levels of *Drp1*, a fission promoting factor, were significantly decreased upon treatment. The *Fis1* mRNA expression profile remained unchanged. To investigate the role of PGC1α alone in regulation of mitochondrial dynamics, *Drp1* and *Mfn1*expression levels were investigated in PGC1α-overexpressing cells. Levels of *Mfn1* expression mRNA) were significantly increased whilst those of *Drp1* were significantly decreased (mRNA and protein) (Fig. 3D and Supplementary Figure 3-S3, B).

This suggested that overexpression of PGC1α was accompanied by a shift of the mitochondrial dynamics profile towards fusion (Fig. 3D). ChIP assay was performed to identify regulatory targets of PGC1α. This revealed significantly increased binding of PGC1α to its own promoter and unexpected binding to the *Drp1* promoter (Fig. 3E). Moreover, PGC1α bound to *Nrf1* and *SOD2* promoters.

**Figure 4:**
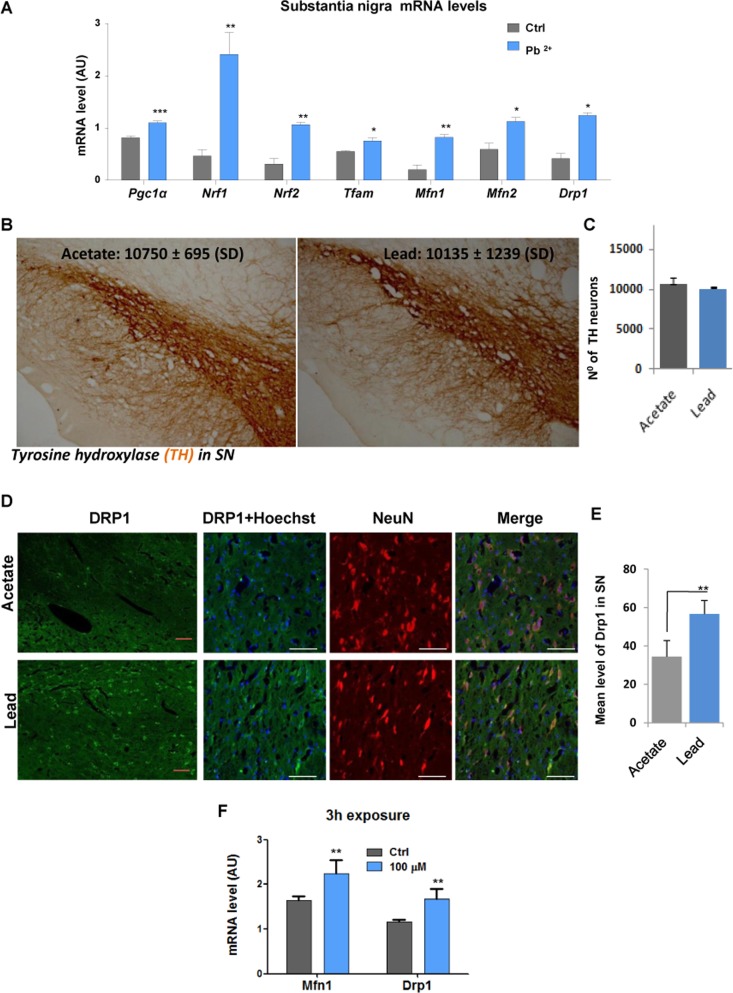
*In vivo* sub-lethallead concentration in drinking water increases the expression of genes controlling mitochondrial biogenesis and dynamics Adult rats received drinking water containing 500 ppm lead acetate for 14 weeks and control animals received water with acetate only. Substantia nigra tissue was isolated and mRNA was extracted and processed for Real time QPCR analysis. (**A**) mRNA levels of *Pgc1α* and its target genes *Nrf1, Nrf2* and *Tfam* as well as*Mfn1, Mfn2*and *Drp1* were analysed by qRT-PCR; (**B**) Immunohistochemistry was performed to detect tyrosine hydroxylase (TH) positive neurons and (**C**) stereological counting of TH-positive neurons in the substantia nigra of rats given drinking water containing 500 ppm lead acetate for 14 weeks. (**D**) Immunohistochemistry of DRP1 and NeuN co-localisation in the ventral mesencephalon neuronal cells. (**E**) Drp1 signal intensity was analysed by confocal laser scanning microscope (Zeiss LSM 7 DUO), using the associated software package (ZEN 2010) and quantified using an Image Analysis Program from Soft Imaging System (analySIS®, Germany). (**F**) *MfnI* and *Drp1* mRNA levels in N27 cells after 3-hours of lead exposure. *P<0.05, **P<0.01, ***P<0.001, n=6 (F, n=3); mean ± SE.

In order to compare the effect of lead on neuronal cells *in vitro* and *in vivo*, tissue samples were obtained from substantia nigra of rats given drinking water containing 500 ppm lead acetate for a period of 14 weeks as well as from control animals given water with acetate only. Real time QPCR analysis showed that *Pgc1α* mRNA levels, as well as levels of its downstream targets (*Nrf1, Nrf2, Tfam)* were significantly increased in the substantia nigra of animals exposed to lead (Fig. 4A). Expression of both mitofusins was also significantly induced by lead (Fig. 4A). Interestingly, unlike N27 dopaminergic neurons exposed to lead for 48-hours *in vitro*, levels of the*Drp1* transcript were increased together with levels of *Mfn1* and *Mfn2* transcipts, after lead exposure *in* vivo, with no decrease in the number of tyrosine hydroxylase-expressing, dopaminergic SN neurons cells (Fig. 4B and C). Since SN tissue used in the experiment was a heterogeneous mixture of different cell types, including neurons, we performed dual fluorescence immunohistochemistry of DRP1 and NeuN (a neuronal specific marker) in the ventral mesencephalon to determine whether the previously observed increase in *Drp1* expression was specific to DA neurons (Fig. 4D). Further analysis confirmed that the expression of DRP1 protein was significantly induced by lead in SN pars compact a neurons only (Fig. 4E). As adult rats are less susceptible to lead toxicity than young rats, and a significant increase of *Drp1* expression was observed *in vivo* at 48-hours, we looked at *Drp1* expression *in vitro* after short-term exposure (Fig. 4F). After 3-hours of lead exposure in N27 cells, an elevation of *Drp1* mRNA levels was observed (*P*<0.01).

### Lead treatment causes a disruption of cellular calcium balance

**Figure 5:**
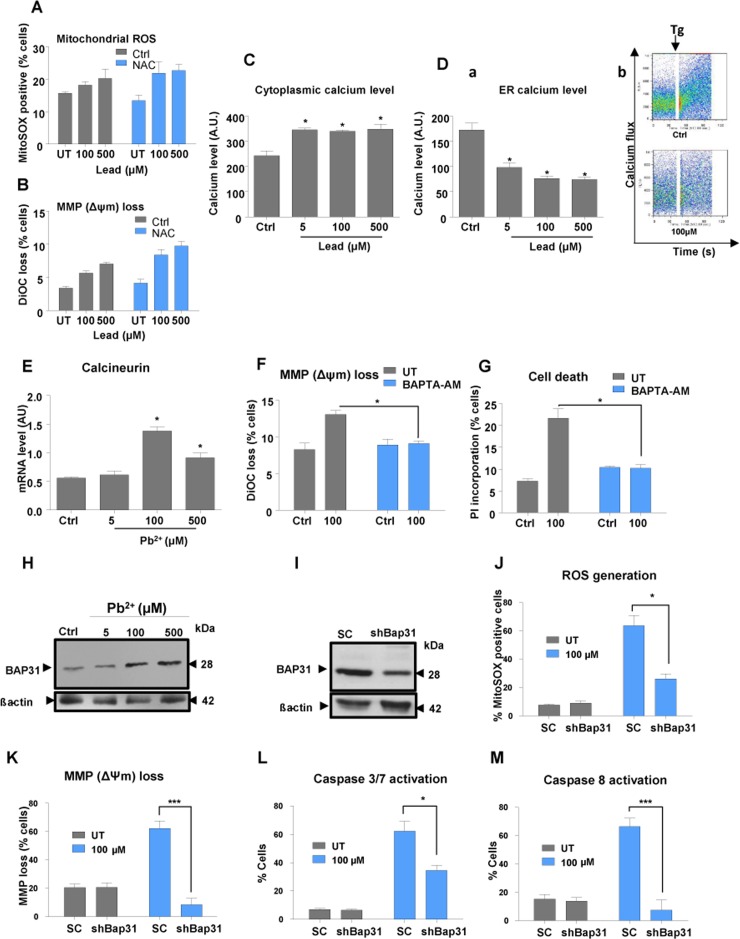
Lead treatment causes a disruption of cellular calcium balance Pre-treatment of N27 cells with 1 mM N-acetylcysteine (NAC) for 1 hour followed by a 48-hours lead-exposure was used to investigate the impact on mitochondrial ROS (**A**) and mitochondrial depolarisation (MMP) (**B**) N27 cells treated with lead for 48-hours were tested for cytoplasmic (**C**) and ER calcium levels (**D**) using thapsigargin (Tg) and Rhod-2 staining followed by flow cytometry analysis (**D**, a and b). Calcineurin transcript levels 48-hours post-lead exposure were investigated using qRT-PCR (**E**). Pre-treatment of N27 cells with 5μM BAPTA-AM for 1 hour followed by a 48-hours lead-exposure was used to investigate the impact of calcium on mitochondrial depolarisation (**F**) and cell death (**G**) following lead treatment. (**H**) Western blot of BAP31 protein levels after 48-hours lead treatment. (**I**) Western blot analysis of BAP31 levels in shBAP31 N27 cells. N27 cells with stable downregulation of BAP31 (shBAP31) were used to investigate the involvement of BAP31 in lead-induced mitochondrial superoxide generation (**J**), mitochondrial depolarisation (MMP) (**K**) and activation of caspases 3/7 (**L**) and 8 (M). *P<0.05, **P<0.01, ***P<0.001, n=3; mean ± SE.

The possible role of ROS in lead-induced cell death in N27 cells was investigated using N-acetylcysteine (NAC), a well-known ROS scavenger. However, NAC pre-treatment of cells had no effect on lead-induced changes in mitochondrial ROS or membrane potential (Fig.5A and B). Therefore, we explored the possibility that changes in intracellular Ca^2+^ levels might mediate the effects of lead, since lead is known to mimic this cation inside the cell and compete with it for binding to Ca^2+^-dependent proteins. Moreover, calcium-regulated proteins have been shown to play a role in control of PGC1α expression [19].

Staining of cells with Fluo4-AM, a cytoplasmic calcium dye, revealed that lead causes an increase of cytosolic Ca^2+^ with depletion within the ER (Fig. 5C and D). Pre-treatment of cells with BAPTA-AM, a cytoplasmic calcium chelator, conferred protection against lead insult (Fig. 5F and G). This finding, together with a lack of response to NAC on cellular susceptibility to lead, implicates a primary role for calcium in the response of dopaminergic neurons to lead. mRNA levels of calcineurin, a modulator of a number of key Ca^2+^ signalling pathways in neurons, were significantly increased on exposure to higher concentrations of lead, with peak expression occurring at 100 μM (Fig. 5E). Calcineurin mRNA levels at 100 μM and 500 μM mirrored those of *Pgc1α* and its target genes *Nrf1* and *Tfam* (Fig 1A-C).

Caspase-8 cleavage of BAP31 at the ER stimulates Ca2+-dependent mitochondrial fission and subsequently enhances pro-apoptotic signals between the ER and mitochondria [6, 20]. Since the protein level of BAP31 increased after exposure to 100μM and 500μM lead (Fig. 5H), we investigated whether BAP31 down-regulation had any neuroprotective effect against lead insult. shRNA delivered by a lentiviral vector was used to knock down ***Bap31*** expression in N27 cells (Fig. 5I). shRNA-***Bap31*** conferred significant protection against lead toxicity (Fig. 5J-M) implying an important role of BAP31 in the response to lead and in calcium pathway regulation.

## DISCUSSION

In this study, we demonstrate for the first time that PGC1α regulates both mitochondrial biogenesis and dynamics in dopaminergic neurons exposed to neurotoxic lead (II) acetate. Despite a 20-fold concentration difference between 5μM and 100μM, the effects of lead acetate on mitochondrial superoxide generation and membrane polarization were comparable. As both of these concentrations exhibited minimal toxicity, they were classified as tolerated. However, at 500 μM, lead had a much more deleterious effect on mitochondrial function and subsequently induced excessive cell death; therefore this concentration was classified as toxic. Interestingly, while most parameters of mitochondrial function remained unaffected by tolerated concentrations of lead, spare respiratory capacity (SRC) was lost at all concentrations, a previously unreported effect of lead. This finding is consistent with the stochastic mitochondrial failure model proposed by Choi, *et al*. [21] to explain the importance of mitochondrial SRC in neurodegeneration. SRC is used here to describe the amount of extra ATP that can be produced by oxidative phosphorylation in case of a sudden increase in energy demand. Depletion of the SRC has been related to a range of pathologies affecting high energy requiring cells such as neurons [21, 22].

As N27 cells were able to tolerate concentrations of lead up to a toxic threshold, we suspected the presence of a protective mechanism, to help cells survive lead insult. PGC1α is a well-established master regulator of mitochondrial metabolism [1] and has previously been reported to play a protective role against a variety of neurodegenerative conditions [23, 24]. PGC1α is also known to respond to ROS by inducing a cellular ROS scavenging program [25]. Due to these diverse functions, we investigated whether PGC1α plays a role in the cellular response to lead exposure. Exposure of cells to 100 μM lead for 48-hours indeed resulted in increased expression of PGC1α and modulation of its downstream targets, including genes regulating mitochondrial biogenesis. Stable down-regulation of *Pgc1α* expression in DA cells increases their vulnerability to lead insult. Thus, we show for the first time that exposure to sub-lethal doses of lead induces low-level stress, which triggers an adaptive response mediated by PGC1α. This supports the hypothesis that PGC1α acts to protect the cells by promoting mitochondrial biogenesis, boosting their function and suppression of ROS [26]. We believe that at low lead levels, slightly increased ROS production, which acts as a retrograde nuclear signal, can improve mitochondrial function with adaptation to the stressor, a phenomenon known as mitohormesis [27].

Short exposure (3-hours) to lead induces a significant increase of *Pgc1α* transcript levels at 100μM and 500μM. However, whereas at 100μM the increase is sustained at 48-hours, at 500 μM, levels return completely to baseline by this time. Hence, lead modulates *Pgc1α* expression in a dose-and time*-*dependent manner. At toxic doses the adaptive response of the cell is initially triggered but becomes overridden by the excessive toxicity, such that it is beyond the homeostatic ability of the cell to counteract the insult. Unexpectedly, overexpression of *Pgc1α* did not confer increased protection against lead, instead, upon treatment with lead, cells showed an increased level of apoptosis compared with wild-type cells. Thus, the role of PGC1α appears to be equivocal: while it helps the cell to tolerate a certain level of toxic insult, prolonged expression to non-physiological levels has a deleterious effect on mitochondrial function and viability of the cells. Ciron et al.[28] have previously shown that sustained expression of PGC1α in dopaminergic neurons leads to neurodegeneration. Therefore, we postulate that PGC1α plays a dual role in response to the neurotoxicant lead. Increased expression at lower concentrations of lead is beneficial as part of an adaptive, mitohormetic response, whereas at higher concentrations, higher expression levels of PGC1α, when sustained for a prolonged period of time, characteristic of chronic exposure, lead to lethal changes in cellular metabolism.

We have observed that changes in the expression of PGC1α result in a structural alteration of mitochondrial filaments. There has been little research investigating the role of PGC1α in orchestrating mitochondrial dynamics. Previously it has been suggested that PGC1α promotes mitochondrial fusion via co-activation of MFN2expression [29]. Moreover, it has recently been shown that PGC1α regulates MFN1. Martin et al.[30]also showed an association between PGC1α/β levels and expression of other fission/fusion genes. However, these authors did not explore the direct interaction between PGC1 proteins and the genes mentioned above. Therefore we investigated the expression of a range of genes known to control mitochondrial dynamics, upon exposure to lead. In this study, we made the novel observation of a PGC1α-induced decrease in DRP1 expression *in vitro*, in addition to increased levels of MFN2 transcript. It appears that the shift of mitochondrial dynamics towards fusion, observed at 100 μM lead, is due to a change in the transcriptional profile of a number of genes controlling this process. A similar effect was seen in cells overexpressing PGC1α, confirming the role of this transcriptional co-activator in the control of mitochondrial dynamics. While *in vivo* data supported the notion of lead increasing the expression of mitofusins (MFN1 and MFN2), and so, promoting fusion, we also observed an increase of Drp1 mRNA. Since substantia nigra obtained from rats, as used in our experiments, is a heterogeneous mixture of different cells with dopaminergic neurons comprising just 1-2% of the total cell population [31], we looked at co-localization of NeuN and DRP1 in samples to exclude the possibility that the global SN mRNA changes observed in our experiment interfere with measurements of the transcriptional state of dopaminergic neurons.

Having confirmed that *Drp1* levels are increased in SN neurons, we sought to match an appropriate exposure duration *in vitro*. Hence, the expression profile of this gene was determined in N27 cells following a short 3-hours exposure to 100 μM lead. *Drp1* expression was increased after this time, but reduced after 48-hours. Hence, this short exposure *in vitro* could be reflecting an early event that happens in DA neurons as protection against lead exposure.

ChIP data revealed that PGC1α binds to the promoter of *Drp1* implying its direct role in regulating the expression of this mitochondrial protein. We propose that as an initial response to the insult *in vivo* and *in vitro*, PGC1α levels increase in response to lead, which promotes mitochondrial biogenesis. This in turn requires an increased expression of proteins regulating mitochondrial dynamics. Tight control of this vital process is essential to maintain balanced intracellular energy distribution and cell viability.

Continuous exposure to tolerated level of lead triggers a protective response which involves a non-excessive expression of genes regulating mitochondrial dynamics with the shift of the balance toward fusion, which has been previously shown to help protect against neurodegeneration [32]. We believe that while the binding of PGC1α to the promoter of *Drp1* at lower concentrations (100μM) results in an increase in the expression of DRP1 to ensure maintenance of fusion-fission balance in the cell, the transcript levels of *Drp1* remain low at high lead concentrations due to the absence of other members of a transcription complex driving expression of this gene, lack of transactivation or presence of an inhibitor, such as e.g. p160^MYB^ [33].

Whilst evidence was obtained that demonstrates PGC1α plays a pivotal role in the response of DA cells to lead, it was unclear the mechanisms by which lead and PGC1α are inter-linked. One possibility was ROS. However, the antioxidant NAC was unable to prevent an increase in lead-induced mitochondrial ROS and subsequent loss of mitochondrial membrane potential. We then focused on calcium, a ubiquitous intracellular secondary messenger. Calcium is of particular interest due to the ability of lead to mimic this ion in the cellular milieu [34]. In the present study, we show that lead causes release of ER calcium stores, increase in cytoplasmic calcium concentrations, which cause disruption of calcium balance leading to mitochondrial dysfunction and DA neuronal cell death. Interestingly, calcineurin, the activity of which depends on Ca^2+^/calmodulin binding, can be activated by low concentrations of free lead while higher concentrations reduce its activity [35]. Our data showed a significant increase in calcineurin transcription levels at a tolerated concentration of lead (100μM) and a clear decline at the toxic concentration (500 μM), an observation consistent with the published data.

Disruption of ER calcium stores has previously been implied in neurodegeneration [36] and has been suggested as one of the mechanisms via which lead causes cognitive dysfunction in neonatal rats [18]. Unlike NAC, pre-treatment of dopaminergic cells with the calcium chelator BAPTA-AM prevented lead-induced mitochondrial membrane potential loss and subsequent cell death, reflecting the upstream role played by this ion in lead toxicity. ER calcium is linked to apoptosis by pro-apoptotic Bap20, the cleavage product of ER-localized BAP31, which mediates mitochondria-ER cross talk through a Ca^2+^-dependent mechanism [37]. Our data showed that stable knockdown of BAP31 significantly decreases activation of caspase-8 as well as caspase 3/7. Caspase-8 cleavage of BAP31 at the ER stimulates Ca^2+^-dependent mitochondrial fission, enhancing cell death [38].

Interestingly, Breckenridge and co-workers [38]have shown that overexpression of Bap20 causes ER Ca^2+^ release and concomitant uptake of Ca^2+^ into mitochondria with subsequent recruitment of DRP1, resulting in dramatic mitochondrial fragmentation. Our data show that the BAP31 protein increases in a dose dependent manner with lead exposure and its knockdown reduces lead-induced initiation of the mitochondrial cell death pathway. This reveals the important role played by this ER protein in disruption of calcium homeostasis in response to lead insult. Together, our data and those of Brekenridge and co-workers strongly suggest the involvement of ER calcium and DRP1-controlled fission in response to lead exposure.

## CONCLUSIONS

**Figure 6:**
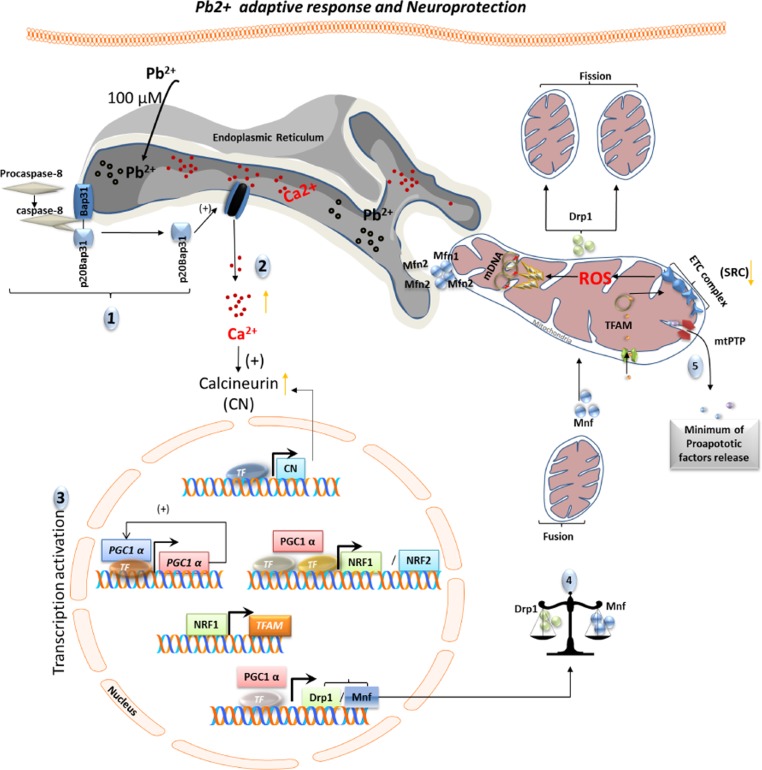
Schematic representation of adaptive response and neuroprotection induced by cell-tolerated levels of Pb2+ Exposure to non-cytotoxic levels of lead (100μM) induces an adaptive biological response. This response involves ER calcium release and requires both caspase-8 activation and BAP31 cleavage (1). Increased cytoplasmic levels of calcium induce expression and activation of calcineurin (2) as well as an increase in PGC1α expression. Subsequently, PGC1α stimulates its own expression and the expression of mitochondrial biogenesis (*NRF1*, *NRF2*, *TMFA*) and dynamics (*DRP1*and *MFN1*) genes (3). A shift of mitochondrial dynamics towards fusion renders neuronal cells more resistant to lead insult (4-5).

We show for the first time that low levels of lead cause disruption of cellular calcium balance in dopaminergic neurons, involving BAP31-mediated ER-mitochondrial crosstalk, which induces an adaptive response mediated by PGC1α. This effect involves an alteration of expression of genes regulating mitochondrial dynamics (shown by us to be controlled by PGC1α). We have also made the novel discovery that PGC1α is directly involved in control of *Drp1* expression (Fig. 6). While beneficial and protective at physiological levels, it appears that long term, chronic stimulation of cells at higher concentrations of lead causes sustained expression of PGC1α at high levels, leading to neurodegeneration via profound remodeling of the mitochondrial network and changes in cellular metabolism. Moreover, any homeostatic imbalance in PGC1α levels (downregulation/overexpression) renders DA cells more susceptible to lead insult. We have also shown that lead causes loss of SRC which renders the cells more susceptible to degeneration when subject to an increased energy demand.

This study provides evidence that the maintenance of a homeostatic PGC1α expression level is essential for protection of neuronal cells against lead insult.

## MATERIALS AND METHODS

### Cell culture and treatments

Rat mesencephalon-derived cell line (1RB_3_AN_27_; hereafter referred to as N27 cells)represent a homogenous population of tyrosine hydroxylase-expressing cells with functional characteristics resembling dopaminergic neurons. N27 cells were maintained in RPMI*-*1640 medium supplemented with 10% FBS, 100 U/ml penicillin and 100 μg/ml streptomycin (Sigma) at 37°C in a 5% CO_2_ humidified atmosphere. Cells were exposed to lead (II) acetate (Sigma) dissolved in distilled water added to the growth medium at 5, 100 and 500 μM for 48-hoursor 3- hours, as indicated.

### Stable cell line production

The pLKO.1-TRC cloning vector was obtained from AddGene. Oligonucleotides containing shRNA were cloned into the vector according to the manufacturer's protocol. For shRNA primers used for PGC1α and BAP31 see Supplementary Material Table 1.

Virions were produced in HEK293T cells, by combining three packaging plasmids (pVSV-G), pRev, pGag.Pol (Invitrogen) with pAdVantage (Promega), and pLKO vector. Water and 2 M CaCl_2_ were added and complexes were mixed with 2× HBS (500 μl) and added to cells drop wise. After an overnight incubation, fresh DMEM medium containing 1 mM sodium butyrate was added. Post-transfection the supernatant was collected, spun down and filter-sterilized. N27 cells were transduced for 8-hours in the presence of polybrene (Sigma). Cells were selected with puromycin 3 days after transduction.

Successful knockdown was confirmed by the reduction in specific mRNA (Fig. 2E and protein, as determined immunocytochemically (Supplementary figure3-S3, A).

### Caspase activity measurement

Caspase activity assays were performed using CaspaTag™ caspase-3/7 or 8 assay kit (Millipore) according to the manufacturer's protocol followed by data collection with FACSCalibur™ (BD Biosciences) and analysis with Cyflogic (CyfLo Ltd.)

### Mitochondrial superoxide measurement

Mitochondrial superoxide levels were measured using MitoSOX™ Red (Invitrogen) according to the manufacturer's guidelines. FACSCalibur™ (BD Biosciences) was used for data collection and Cyflogic (CyfLo Ltd.) for the analysis.

### Mitochondrial membrane depolarisation

Cells were harvested as described above and resuspended in 150 μl of PBS containing 40 nM 3,3-dihexaoxacarbocyanine iodide (DiOC6) and PI (50 μM) for 40 minutes at 37°C. Cells were then analysed using FACSCalibur™.

### RNA extraction from cells

Total RNA extraction was performed using QiaShredder columns and the RNeasy extraction kit (Qiagen).

### Animals and surgery

Twenty male albino Wistar rats (250-270 g) were kept at constant room temperature of 22 ± 1 °C and relative humidity (60%) with a 12-h light-dark cycle (8:00 a.m.-8:00 p.m.), with free access to food and water containing either sodium lead acetate (500 ppm) or sodium acetate for 14 weeks. Then, half of the animals in each group were killed by decapitation (RT-PCR experiments) or by perfusion (immunohisto-chemistry experiments).

### Fluorescence immunohistochemistry

Thaw-mounted 20-μm coronal sections were cut on a cryostat at −15 °C and mounted in gelatin-coated slides. For double-labelling of DRP1 with NeuN, sections were blocked with PBS containing 1% normal goat serum and horse serum (Vector) for 1 h. The slides were washed three times in PBS and then incubated overnight at 4°C with either rabbit-derived anti-DRP1 (1:300; Cell Signalling) or mouse-derived anti-NeuN (1:1000; Millipore) diluted in PBS containing 1% normal goat/horse serum and 0.25% Triton X-100. Sections were incubated with goat anti-rabbit secondary antibody conjugated to fluorescein (1:200, for DRP1; Vector) and horse anti-mouse secondary antibody conjugated to Texas Red (1: 200, for NeuN; Invitrogen) for 1 h. Nuclei were counterstained with Hoechst (1 μg ml^−1^, Molecular Probes). Fluorescence images were acquired using a confocal laser scanning microscope (Zeiss LSM 7 DUO) and processed using the associated software package (ZEN 2010). Signal intensity was quantified using an Image Analysis Program from Soft Imaging System (analySIS®, Germany).

### Stereological counting of TH-positive neurons in SN

The number of TH-positive neurons in the SN was estimated using a fractionator sampling design. Counts were made at regular predetermined intervals (x = 150 μm and y = 200 μm) within each section. An unbiased counting frame of known area (40 × 25 μm = 1000 μm^2^) was superimposed on the tissue section image under a 100× oil immersion objective. Therefore, the area sampling fraction is 1000/(150 × 200) = 0.033. The entire z-dimension of each section was sampled; hence, the section thickness sampling fraction was 1. In all animals, 20-μm sections, each 100 μm apart, were analyzed; thus, the fraction of sections sampled was 20/100 = 0.20. The number of neurons in the analyzed region was estimated by multiplying the number of neurons counted within the sample regions by the reciprocals of the area sampling fraction and the fraction of section sampled[39].

### RNA extraction from tissue

Rat substantia nigra were dissected with reference to a standard rat brain atlas (Paxinos and Wilson, 6^th^ Edition, Elsevier), immediately snap-frozen in liquid nitrogen and stored at −80C. Total RNA was extracted using the phenol-chloroform method. Ice-cold Trizol reagent (Life Technologies) was added to 25-50mg of sample according to the manufacturer's instructions. Brain tissue kept on ice was homogenized using a tissue homogenizer (OMNI international).

### Real-time PCR

Five μg RNA was used to synthesize cDNA using the ThermoScript™ RT-PCR first strand synthesis system (random hexamer method) according to manufacturer's instructions (Invitrogen). Platinum SYBR Green qPCR Super Mix-UDG (Invitrogen) was used for qPCR analysis. Primers were designed using Primer Blast (supplementary data, table 1). Real time PCR reactions were performed using Stratagene Mx3000P instrument. MxPro Software (Stratagene) was used to obtain Ct values. Transcript levels relative to GAPDH were determined using the standard curve method.

### Mitochondrial function assessment

The Seahorse XF analyzer was used according to manufacturer's guidelines (Seahorse Bioscience). OCR values were measured by XF24 extracellular flux analyzer.

### Immunocytochemistry

Cells were stained with MitoTracker Red (Molecular probes) according to the manufacturer's protocol, fixed with 4% paraformaldehyde and blocked in 10mM HEPES, 0.3% TX-100, 3% BSA, pH 7.4 for 1 hour at RT. Coverslips were subsequently incubated with PGC1α antibody (overnight; Everest) and AlexaFluor 488 anti-goat IgG secondary antibody (RT, 1 h; Molecular Probes). Nuclei were counterstained with Hoechst (Molecular Probes).

### Western blot analysis

Cells were homogenized on ice in lysis buffer (1 M Tris-HCL, 2.5 M NaCl, glycerol, 0.5 M β-glycerophosphate, Tween 20 and Nonidet P40). The lysate was cleared by centrifugation at 900 g for 10 min at 4 °C. 50-120 μg of proteins were run on a 10% SDS-PAGE gel and transferred to nitrocellulose membranes (GE healthcare). Membranes were blocked with 5% non-fat dry milk dissolved in PBS with 0.01% Tween 20 (Sigma). Incubation with primary antibodies was performed overnight at 4°C (see supplementary materials for the antibody list). Peroxidase activity was developed using enhanced chemiluminescence (ECL).

### Chromatin immunoprecipitation

N27 cells were treated and fixed with formaldehyde and quenched with glycine. Chromatin immunoprecipitation of samples was performed as described previously [40] using PGC1α antibody, followed by Q-RT-PCR analysis. An irrelevant goat antibody (IgG) was used as a negative control, to control for the possibility of non-specific clumping of chromatin. C_t_ values were normalised according to the following equation: ∆C_t_[normalised ChIP] = (C_t_[ChIP] – (C_t_[input] – log_2_ (0.1))). The % input was calculated with the following equation: % input = 2^(−∆Ct[normalised ChIP])^. For the list of target promoter primer pairs see supplementary materials. PGC-1α binding sites were all within promoter regions, defined as ±3 kb from a transcriptional start site (TSS). (Please see supplementary figure 3-S3, C).

## SUPPLEMENTARY MATERIAL TABLE AND FIGURES


