# Erratum: PGC-1α controls mitochondrial biogenesis and dynamics in lead-induced neurotoxicity

**DOI:** 10.18632/aging.100837

**Published:** 2015-09-04

**Authors:** Aleksandra Dabrowska, Jose Luis Venero, Ryota Iwasawa, Mohammed-khair Hankir, Sunniyat Rahman, Alan Boobis, Nabil Hajji

**Aging (Albany NY) 2015; 7(9)**: **629-647**.

PMCID: PMC 4600622 PMID: 26363853

In this Article, the error was introduced into Figure 5 during the production process. The image of Figure 4 was mistakenly posted in Figure 5 making Figures 4 and 5 the same in PDF version with different captions.

The correct PDF copy of this Article is found at: http://www.impactaging.com/papers/v7/n9/pdf/100790.pdf.

