# Erratum: PGC-1α controls mitochondrial biogenesis and dynamics in lead-induced neurotoxicity

**DOI:** 10.18632/aging.100955

**Published:** 2016-04-23

**Authors:** Aleksandra Dabrowska, Jose Luis Venero, Ryota Iwasawa, Mohammed-khair Hankir, Sunniyat Rahman, Alan Boobis, Nabil Hajji

**Aging (Albany NY) 2015; 7(9)**: **629-647**.

PMCID: PMC 4600622 PMID: 26363853

In this Article, the additional author Aine Brigette Henley is added to this manuscript:

http://www.impactaging.com/papers/v7/n9/pdf/100790.pdf. The updated authors’ list with affiliations is provided here:

**Aleksandra Dabrowska^1^, Jose Luis Venero^2^, Ryota Iwasawa^1^, Mohammed-khair Hankir^3^, Sunniyat Rahman^1^, Aine Brigette Henley^4^, Alan Boobis^1^, and Nabil Hajji^1^**

^1^ Imperial College London, Centre for Pharmacology and Therapeutics, Department of Medicine, London, United Kingdom;

^2^ Departamento de Bioquímica y Biología Molecular, Facultad de Farmacia, Universidad de Sevilla, C/Prof. García González, Sevilla, Spain;

^3^ Integrated Research and Treatment Centre for Adiposity Diseases, Department of Medicine, University of Leipzig, Leipzig, Germany:

^4^ Research Centre for Optimal Health, Faculty of Science and Technology, University of Westminster, London, United Kingdom

