# Supplementary material for: PGC-1α controls mitochondrial biogenesis and dynamics in lead-induced neurotoxicity
Source: Aging (Albany NY). 2015 Sep 4;7(9):629–43. doi: 10.18632/aging.100790 (PMC4600622; doi:10.18632/aging.100790)
Supplement: Supplementary file 1 [file aging-07-629-s001.pdf]

SUPPLEMENTARY MATERIAL

Supplementary Material Table1.

Target	Primer sequence
Promoter DRP1 F	CAGATTCACGGACCCAGCTT
Promoter DRP1 R	CAAAGGCTGTCGGGAGATGT
Promoter PGC1α F	GGACCTTTGCTGTTTGCCTG
Promoter PGC1 α R	GCCACCAACTCTAAACCGGA
Promoter SOD2 F	CCTGGCCTTCTAATCCCGAC
Promoter SOD2 R	CCCTGAGGTTAGGGTTGCTG
MFN1 F	CCATCACTGCGATCTTCGGCCA
MFN1 R	CAGCGAGCTTGTTTCTGTAGCCCT
MFN2 F	CTCTGTGCTGGTTGACGAGT
MFN2 R	AGCGGTCAGACATGTTTCGT
FIS1 F	GTAGGGTTACATGGATGCCCAGAGA
FIS1 R	GGCAACAGCTCCTCCAGCAG
DRP1 F	GGTGGAATTGGAGATGGTGGTCGA
DRP1 R	TTCGTGCAACTGGAACTGGCACA
PGC1α F	ACTGAGCTACCCTTGGGATG
PGC1α R	TAAGGATTTCGGTGGTGACA
TFAM F	GTCGCGTGGGACAAACCGGA
TFAM R	CACATCTCGACCCCCGTGCG
GAPDH F	GGGGCTCTCTGCTCCTCCCTG
GAPDH R	CGGCCAAATCCGTTCACACCG
CaNB F	TCCAACCGAGACTCCGCCCG
CaNB R	GACGGCGGCTTTGCTGTCCG
NRF1 F	TTACT TGCTGTGGCTGATGG
NRF1 R	CCTCTGATGCTTGCGTCGTCT
NRF2 F	CAGAGCAAGTGACGAGATGG
NRF2 R	CCGAAATGTTGAGTGTGGTG
Scramble shRNAF	CCGGCCTAAGGTTAAGTCGCCCTCGCTCT AGCGAGGGCGACTTAACCTTAGGTTTTTG
Scramble shRNA R	AATTCAAAAACCTAAGGTTAAGTCGCCCT CGCTCTAGCGAGGGCGACTTAACCTTAGG
shRNABAP31 F	CCGGGACGCCTGGTGACTCTCATTTCTCG AGAAATGAGAGTCACCAGGCGTCTTTTTG
shRNA BAP31 R	AATTCAAAAAGACGCCTGGTGACTCTCAT TTCTCGAGAAATGAGAGTCACCAGGCGTC
shRNAPGC1 α F	CCCTGCCATTGTTAAGAGACC
shRNAPGC1 α R	TGCTGCTGTTCCTGTTTTC

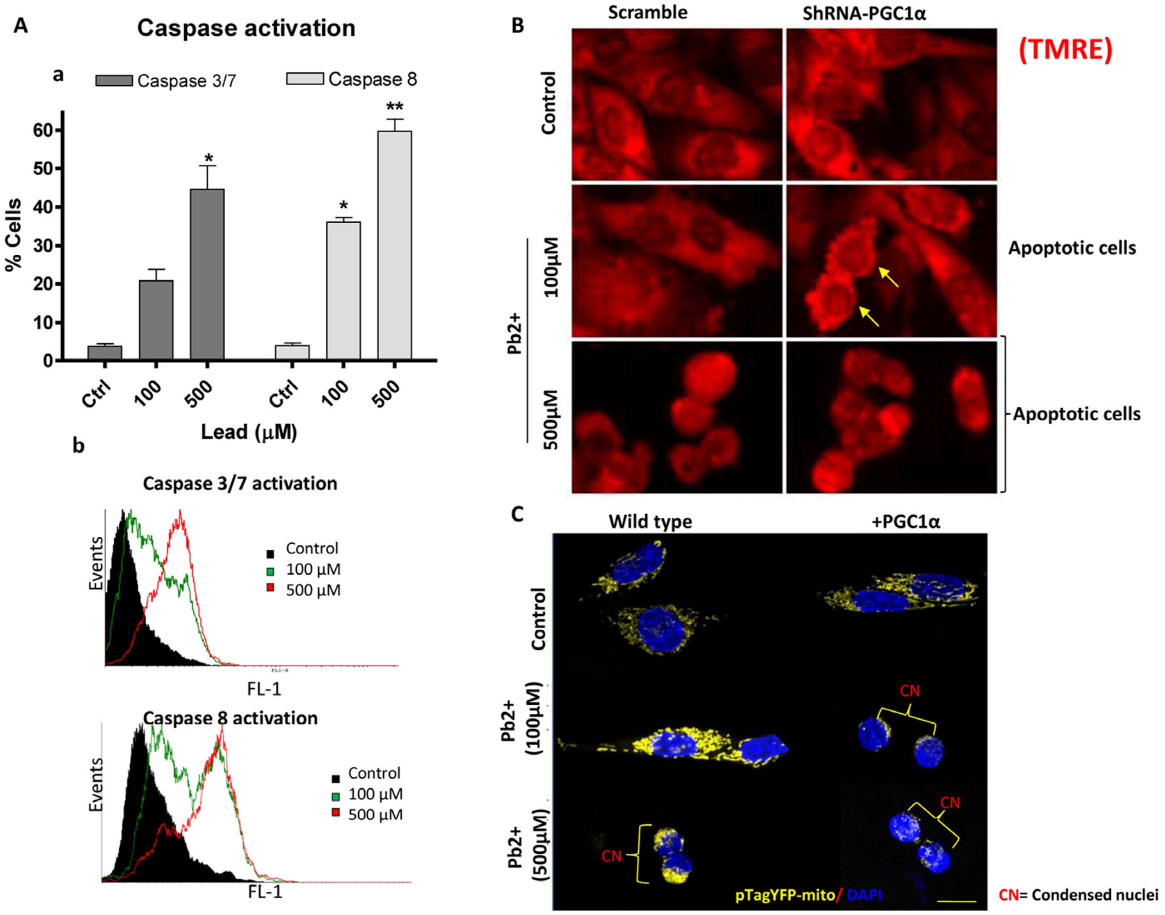

Supplementary Figure 1-S1. Assessment of apoptotic cell death induced by lead.(**A**-a) FCAS analysis of caspases3/7 and caspase 8 activation in N27 cells exposed to 100μM and 500μM lead. (**A**-b) Representative FACS histogram of caspases3/7 and caspase 8 activation. (**B**) Confocal mycroscopy analysis of morphological features of apoptotic cells in both scramble and shRNA-PGC1α N27 cells. Cells were exposed to 100μM and 500μM lead, pre-stained with TMRE and immunohistochemistry assay was performed. (**C**) Wild type and overexpressed PGC1α cells were transfected with pTagYFP-mito and the cells were treated with lead (100μM and 500μM) followed by immunohistochemistry assay. Cell nucleus was stained with DAPI. CN (Condensed nuclei). *P<0.05, **P<0.01, n=3; mean ± SE.

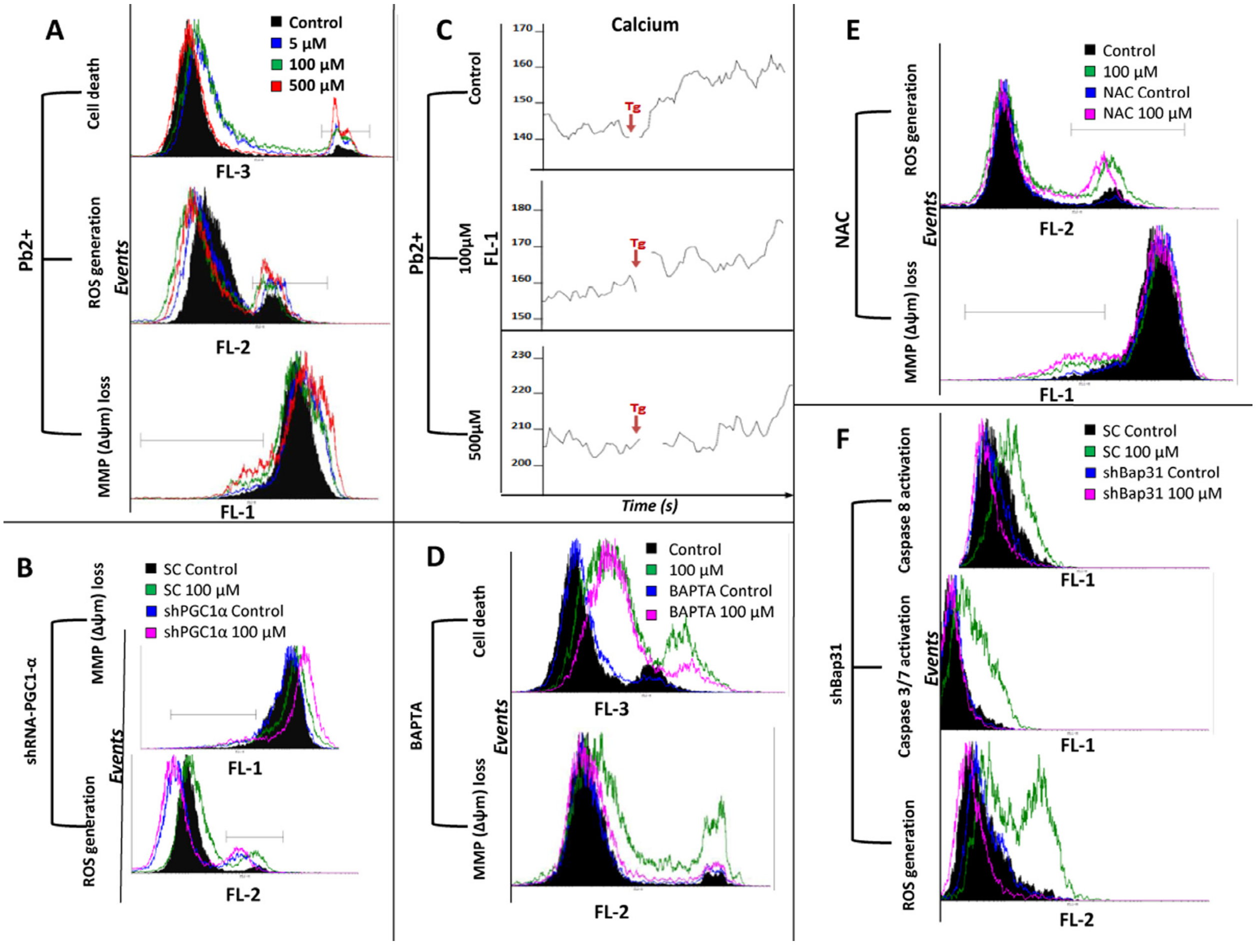

Supplementary Figure 2-S2. Representative FACS analysis histograms of N27 cells exposed to lead.(**A**) Analysis of mitochondrial depolarisation [MMP (Δψm) loss], mitochondrial ROS generation and cell death in N27 cells treated with lead for 48-hours. (**B**) Scramble and shRNA PGC1-α cells were treated with 100µM of lead for 48-hours and mitochondrial ROS generation and MMP (Δψm) loss were analysed by FACS. (**C**) N27 cells treated with lead for 48-hours were tested for calcium release using thapsigargin (Tg) and Rhod-2 staining followed by flow FACS analysis. (**D**) Pre-treatment of N27 cells with 5µM BAPTA-AM for 1 hour followed by a 48h lead-exposure and FACS analysis of MMP (Δψm) loss and cell death.(**E**) Pre-treatment of N27 cells with 1 mM N-acetylcysteine (NAC) for 1 hour followed by a 48h lead-exposure and later mitochondrial ROS and MMP (Δψm) FACS analysis.(**F**) N27 cells with stable downregulation of BAP31 (shBAP31) were used to investigate the involvement of BAP31 in lead-induced mitochondrial ROS and caspases 3/7 and 8 activation.

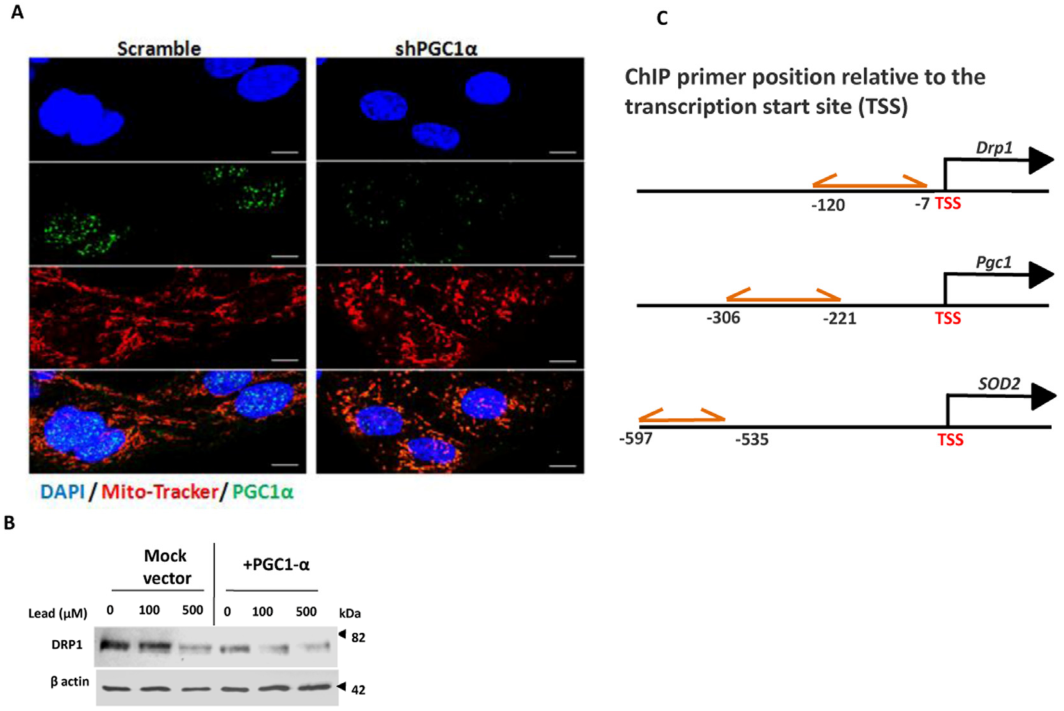

Supplementary Figure 3-S3. Assessment of PGC-1α and Drp-1 protein levels plus ChIP primer position relative to the transcription start site (TSS). (**A**) Immunocytochemistry analysis of PGC-1α protein level in PGC-1α knock-down (sh-PGC1α) cells compared with the scramble cells. (**B**) Western blot analysis of transfected cells with PGC-1α or mock vector treated with lead (100µM or 500µM). Beta-actin is used as a loading control. (**C**) ChIP primer position relative to the transcription start site (TSS).
